# (4b*S*,8a*S*)-1-Isopropyl-4b,8,8-trimethyl-4b,5,6,7,8,8a,9,10-octa­hydro­phenan­thren-2-yl benzoate

**DOI:** 10.1107/S1600536814015827

**Published:** 2014-07-11

**Authors:** Radouane Oubabi, Aziz Auhmani, My Youssef Ait Itto, Abdelwahed Auhmani, Jean-Claude Daran

**Affiliations:** a Laboratoire de Synthése Organique et Physico-Chimie Moléculaire, Département de Chimie, Faculté des Sciences Semlalia, BP 2390 Marrakech 40000, Morocco; b Laboratoire de Chimie de Coordination, 205 route de Narbonne, 31077 Toulouse Cedex 04, France

**Keywords:** crystal structure

## Abstract

The title compound, C_27_H_34_O_2_, was hemisynthesized through direct benzoyl­ation of the naturally occurring meroterpene totarol. The central fused six-membered ring has a half-chair conformation, whereas the terminal six-membered ring displays a chair conformation. The dihedral angle between the fused benzene ring and the benzoyl benzene ring is 73.05 (14)°. The *S*,*S* chirality of the mol­ecule is consistent with the synthetic pathway, and confirmed by the refinement of the Flack parameter.

## Related literature   

For the synthesis and biological activity of totarol [systematic name: (4b*S*,8a*S*)-4b,8,8-trimethyl-1-propan-2-yl-5,6,7,8a,9,10-hexa­hydro­phenanthren-2-ol], see: Short & Stromberg (1937 ▶); Barrero *et al.* (2003 ▶); Haraguchi *et al.* (1996 ▶); Bernabeu *et al.* (2002 ▶); Marcos *et al.* (2003 ▶); Tacon *et al.* (2012 ▶). For conformational analysis and absolute configuration determination, see: Cremer & Pople (1975 ▶); Flack (1983 ▶); Flack & Bernardinelli (2000 ▶); Parsons *et al.* (2013 ▶); Spek (2009 ▶). For related structures, see: Zeroual *et al.* (2008 ▶); Oubabi *et al.* (2014 ▶); Pettit *et al.* (2004 ▶).
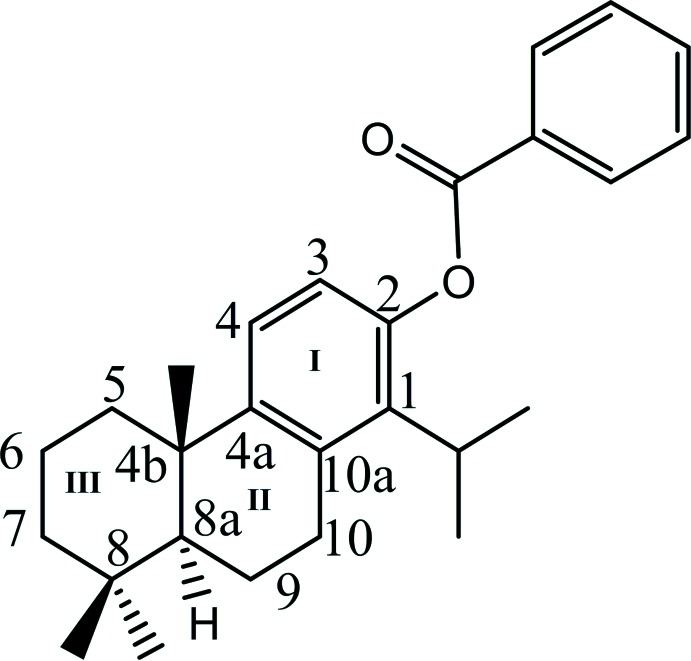


## Experimental   

### 

#### Crystal data  

C_27_H_34_O_2_
*M* *_r_* = 390.54
Monoclinic, 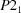

*a* = 7.7369 (3) Å
*b* = 7.2079 (4) Å
*c* = 20.2499 (9) Å
β = 99.816 (4)°
*V* = 1112.74 (9) Å^3^
*Z* = 2
Cu *K*α radiation
μ = 0.55 mm^−1^
*T* = 180 K
0.50 × 0.25 × 0.07 mm

#### Data collection  

Agilent Xcalibur (Eos, Gemini ultra) diffractometer
Absorption correction: multi-scan (*CrysAlis PRO*; Agilent, 2012 ▶) *T*_min_ = 0.689, *T*_max_ = 1.0
9197 measured reflections
3116 independent reflections
2926 reflections with *I* > 2σ(*I*)
*R*_int_ = 0.033
θ_max_ = 60.8°

#### Refinement  

*R*[*F* ^2^ > 2σ(*F* ^2^)] = 0.038
*wR*(*F* ^2^) = 0.095
*S* = 1.04
3116 reflections
267 parameters
1 restraint
H-atom parameters constrained
Δρ_max_ = 0.13 e Å^−3^
Δρ_min_ = −0.21 e Å^−3^
Absolute structure: Flack *x* determined using 1138 quotients [(*I* ^+^)−(*I* ^−^)]/[(*I* ^+^)+(*I* ^−^)] (Parsons *et al.*, 2013 ▶)
Absolute structure parameter: −0.11 (17)

### 

Data collection: *CrysAlis PRO* (Agilent, 2012 ▶); cell refinement: *CrysAlis PRO*; data reduction: *CrysAlis PRO*; program(s) used to solve structure: *SIR97* (Altomare *et al.*, 1999 ▶); program(s) used to refine structure: *SHELXL2013* (Sheldrick, 2008 ▶); molecular graphics: *ORTEP-3 for Windows* (Farrugia, 2012 ▶); software used to prepare material for publication: *SHELXL2013*.

## Supplementary Material

Crystal structure: contains datablock(s) I, New_Global_Publ_Block. DOI: 10.1107/S1600536814015827/bh2501sup1.cif

Structure factors: contains datablock(s) I. DOI: 10.1107/S1600536814015827/bh2501Isup2.hkl

CCDC reference: 1012402

Additional supporting information:  crystallographic information; 3D view; checkCIF report

## Figures and Tables

**Fig. 1.**
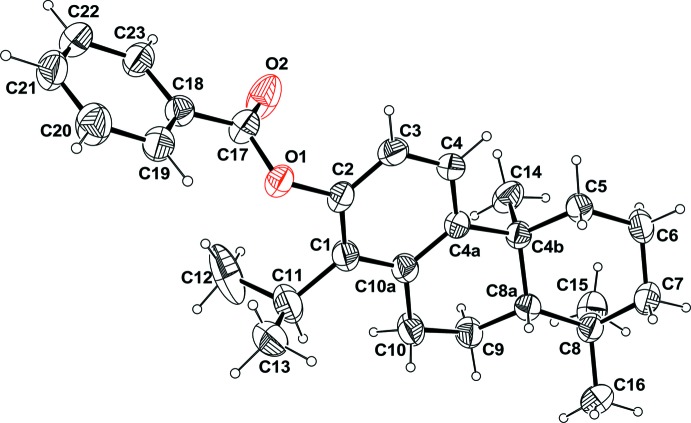
Molecular view of the title compound with ellipsoids for non-H atoms drawn at the 50% probability level. H atoms are represented as small circle of arbitrary radii.

## supplementary crystallographic information

### S1. Results and discussion

Totarol is a naturally produced terpenoid isolated from several plants such as *Podocarpus totara* (Short & Stromberg, 1937) and *Tetra­clinis articulata* (Barrero *et al.*, 2003). It has been attracting great inter­est because of its biological properties ranging from anti­microbial (Haraguchi *et al.*, 1996), anti-oxidant (Bernabeu *et al.*, 2002), anti-inflammatory, analgesic, anti-tumoral (Marcos *et al.*, 2003) to anti-plasmodial (Tacon *et al.*, 2012).

In our ongoing studies on the synthesis of totarol derivatives of potential inter­est, we carried out the reaction of totarol with benzoyl chloride in pyridine, which provides the expected benzoyl­ated product, (4b*S*,8a*S*)-1-iso­propyl-4b,8,8-tri­methyl-4b,5,6,7,8,8a,9,10-o­cta­hydro­phenanthren-2-yl benzoate, as colorless crystals in 92% yield. Its structure was characterized by mass and NMR spectroscopy, and was fully confirmed by an X-ray single crystal structure analysis.

This compound is built up from three fused six-membered rings, an unsaturated benzene ring (I) and two saturated rings (II) and (III) (Fig. 1). The central saturated ring (II) has a half chair conformation with puckering parameters *Q* = 0.527 (3) Å, θ= 48.6 (3)° and φ= 128.9 (4)° (Cremer & Pople, 1975), whereas the second saturated six-membered ring, (III), displays a chair conformation with puckering parameters *Q* = 0.546 (3) Å, θ= 175.8 (3)° and φ= 301 (4)°. Similar conformation for the three fused rings has been reported previously with hydroxyl substituent or methyl acetate in place of the benzoate of the title compound (Zeroual *et al.*, 2008; Oubabi *et al.*, 2014), and with either an hydroxyl or a meth­oxy substituent on the central ring (Pettit *et al.*, 2004).

The 4b*S*,8a*S* absolute configuration is deduced from the synthetic pathway. Although the Flack (Flack, 1983; Flack & Bernardinelli, 2000; Parsons *et al.*, 2013) and Hooft parameters (Spek, 2009) display large standard deviations, their values, -0.11 (17) and 0.09 (15), confirmed the expected absolute configuration.

### S2.1. Synthesis and crystallization

A solution of totarol (110 mg, 0.384 mmol) in benzoyl chloride (3 mL) and pyridine (20 mL) was refluxed for 24 hours. After cooling, the mixture was acidified with HCl (1N solution), and then extracted with ether (3×20 mL). The organic layer was washed with water, dried on anhydrous Na_2_SO_4_ and then evaporated under reduced pressure. The obtained residue was chromatographed on a silica gel column using hexane and ethyl acetate (97/3) as eluent, to give the title compound in 92 % yield. X-ray quality crystals were obtained by slow evaporation from a petroleum ether solution of the title compound.

### S2.2. Refinement

Crystal data, data collection and structure refinement details are summarized in Table 1.

### S3. Refinement

All H atoms were fixed geometrically and treated as riding with C—H = 0.99 Å (methylene), 0.98 Å (methyl), 1.0 Å (methine) and 0.95 Å (aromatic), and with *U*_iso_(H) = 1.2*U*_eq_(CH, CH_2_, aromatic) or *U*_iso_(H) = 1.5*U*_eq_(CH_3_). Owing to physical limitations on the diffractometer, the maximum value of θ used was 60.8° for a complete data set, resulting in the value of sin(θ_max_)/λ less than 0.6 and, consequently, a low fraction of unique reflections (0.834) measured at the best achieved resolution (θ=67.7°).

### Crystal data

**Table:**

C_27_H_34_O_2_	*F*(000) = 424
*M**_r_* = 390.54	*D*_x_ = 1.166 Mg m^−^^3^
Monoclinic, *P*2_1_	Cu *K*α radiation, λ = 1.54184 Å
*a* = 7.7369 (3) Å	Cell parameters from 4294 reflections
*b* = 7.2079 (4) Å	θ = 4.4–60.4°
*c* = 20.2499 (9) Å	µ = 0.55 mm^−^^1^
β = 99.816 (4)°	*T* = 180 K
*V* = 1112.74 (9) Å^3^	Flattened, colourless
*Z* = 2	0.50 × 0.25 × 0.07 mm

### Data collection

**Table:**

Agilent Xcalibur (Eos, Gemini ultra) diffractometer	3116 independent reflections
Radiation source: Enhance Ultra (Cu) X-ray Source	2926 reflections with *I* > 2σ(*I*)
Mirror monochromator	*R*_int_ = 0.033
Detector resolution: 16.1978 pixels mm^-1^	θ_max_ = 60.8°, θ_min_ = 4.4°
ω scans	*h* = −8→8
Absorption correction: multi-scan (*CrysAlis PRO*; Agilent, 2012)	*k* = −8→7
*T*_min_ = 0.689, *T*_max_ = 1.0	*l* = −22→22
9197 measured reflections	

### Refinement

**Table:**

Refinement on *F*^2^	Secondary atom site location: difference Fourier map
Least-squares matrix: full	Hydrogen site location: inferred from neighbouring sites
*R*[*F*^2^ > 2σ(*F*^2^)] = 0.038	H-atom parameters constrained
*wR*(*F*^2^) = 0.095	*w* = 1/[σ^2^(*F*_o_^2^) + (0.0501*P*)^2^ + 0.1662*P*] where *P* = (*F*_o_^2^ + 2*F*_c_^2^)/3
*S* = 1.04	(Δ/σ)_max_ < 0.001
3116 reflections	Δρ_max_ = 0.13 e Å^−^^3^
267 parameters	Δρ_min_ = −0.21 e Å^−^^3^
1 restraint	Absolute structure: Flack x determined using 1138 quotients [(*I*^+^)-(*I*^-^)]/[(*I*^+^)+(*I*^-^)] (Parsons *et al.*, 2013)
0 constraints	Absolute structure parameter: −0.11 (17)
Primary atom site location: structure-invariant direct methods	

### Fractional atomic coordinates and isotropic or equivalent isotropic displacement parameters (Å^2^)

**Table:**

	*x*	*y*	*z*	*U*_iso_*/*U*_eq_	
O1	1.0825 (2)	0.2023 (3)	0.33409 (8)	0.0388 (5)	
O2	1.2632 (2)	0.2265 (4)	0.25828 (10)	0.0661 (7)	
C1	0.8685 (3)	0.4403 (4)	0.29484 (14)	0.0413 (7)	
C2	0.9421 (3)	0.2698 (4)	0.28549 (13)	0.0359 (6)	
C3	0.8814 (3)	0.1570 (4)	0.23162 (13)	0.0385 (7)	
H3	0.9338	0.0395	0.2270	0.046*	
C4	0.7432 (3)	0.2179 (4)	0.18449 (13)	0.0374 (6)	
H4	0.7019	0.1416	0.1468	0.045*	
C4A	0.6625 (3)	0.3879 (4)	0.19052 (12)	0.0319 (6)	
C4B	0.5137 (3)	0.4506 (4)	0.13429 (12)	0.0330 (6)	
C5	0.4014 (3)	0.2837 (4)	0.10522 (14)	0.0405 (7)	
H5A	0.4730	0.2022	0.0812	0.049*	
H5B	0.3675	0.2113	0.1426	0.049*	
C6	0.2361 (4)	0.3401 (5)	0.05730 (15)	0.0462 (8)	
H6A	0.2691	0.4028	0.0178	0.055*	
H6B	0.1676	0.2279	0.0415	0.055*	
C7	0.1239 (3)	0.4697 (4)	0.09132 (14)	0.0425 (7)	
H7A	0.0835	0.4024	0.1285	0.051*	
H7B	0.0188	0.5050	0.0587	0.051*	
C8	0.2197 (3)	0.6458 (4)	0.11908 (13)	0.0390 (7)	
C8A	0.3948 (3)	0.5887 (4)	0.16448 (12)	0.0334 (6)	
H8A	0.3585	0.5209	0.2030	0.040*	
C9	0.5059 (4)	0.7494 (4)	0.19617 (15)	0.0431 (7)	
H9A	0.5673	0.8071	0.1623	0.052*	
H9B	0.4290	0.8445	0.2113	0.052*	
C10	0.6400 (4)	0.6854 (4)	0.25540 (15)	0.0470 (7)	
H10A	0.5817	0.6768	0.2952	0.056*	
H10B	0.7328	0.7810	0.2649	0.056*	
C10A	0.7261 (3)	0.5000 (4)	0.24594 (13)	0.0379 (6)	
C11	0.9377 (5)	0.5598 (5)	0.35593 (17)	0.0653 (11)	
H11	0.8681	0.6774	0.3505	0.078*	
C12	1.1296 (6)	0.6161 (8)	0.36183 (19)	0.0941 (16)	
H12A	1.2045	0.5079	0.3745	0.141*	
H12B	1.1568	0.7127	0.3961	0.141*	
H12C	1.1508	0.6641	0.3186	0.141*	
C13	0.9041 (5)	0.4715 (8)	0.42098 (18)	0.0814 (14)	
H13A	0.7788	0.4440	0.4174	0.122*	
H13B	0.9404	0.5574	0.4583	0.122*	
H13C	0.9716	0.3562	0.4290	0.122*	
C14	0.6051 (3)	0.5319 (5)	0.07852 (14)	0.0494 (8)	
H14A	0.6672	0.6460	0.0947	0.074*	
H14B	0.5170	0.5598	0.0390	0.074*	
H14C	0.6892	0.4414	0.0666	0.074*	
C15	0.2425 (4)	0.7775 (5)	0.06206 (17)	0.0540 (8)	
H15A	0.2917	0.7092	0.0277	0.081*	
H15B	0.3222	0.8783	0.0796	0.081*	
H15C	0.1283	0.8293	0.0423	0.081*	
C16	0.1036 (4)	0.7443 (6)	0.16232 (18)	0.0629 (10)	
H16A	−0.0121	0.7680	0.1355	0.094*	
H16B	0.1580	0.8622	0.1785	0.094*	
H16C	0.0907	0.6655	0.2006	0.094*	
C17	1.2417 (3)	0.1844 (4)	0.31324 (14)	0.0413 (7)	
C18	1.3781 (3)	0.1078 (4)	0.36592 (13)	0.0366 (6)	
C19	1.3442 (4)	0.0440 (5)	0.42702 (13)	0.0424 (7)	
H19	1.2289	0.0526	0.4371	0.051*	
C20	1.4771 (4)	−0.0320 (5)	0.47326 (15)	0.0546 (8)	
H20	1.4535	−0.0762	0.5150	0.066*	
C21	1.6455 (4)	−0.0433 (5)	0.45829 (16)	0.0532 (8)	
H21	1.7372	−0.0958	0.4899	0.064*	
C22	1.6800 (4)	0.0207 (5)	0.39825 (16)	0.0513 (8)	
H22	1.7957	0.0133	0.3886	0.062*	
C23	1.5477 (3)	0.0959 (5)	0.35175 (14)	0.0457 (7)	
H23	1.5721	0.1395	0.3100	0.055*	

### Atomic displacement parameters (Å^2^)

**Table:**

	*U*^11^	*U*^22^	*U*^33^	*U*^12^	*U*^13^	*U*^23^
O1	0.0294 (9)	0.0476 (12)	0.0387 (9)	0.0043 (9)	0.0037 (7)	0.0049 (9)
O2	0.0380 (11)	0.110 (2)	0.0515 (13)	0.0043 (13)	0.0107 (9)	0.0304 (14)
C1	0.0373 (14)	0.0389 (17)	0.0442 (15)	0.0012 (13)	−0.0026 (12)	−0.0076 (13)
C2	0.0268 (12)	0.0423 (18)	0.0375 (14)	0.0014 (13)	0.0026 (10)	0.0028 (13)
C3	0.0338 (13)	0.0370 (17)	0.0453 (15)	0.0043 (13)	0.0085 (11)	−0.0029 (13)
C4	0.0334 (13)	0.0381 (17)	0.0406 (14)	0.0010 (13)	0.0064 (11)	−0.0082 (13)
C4A	0.0281 (13)	0.0335 (16)	0.0342 (14)	−0.0025 (11)	0.0059 (10)	−0.0010 (11)
C4B	0.0303 (13)	0.0361 (16)	0.0324 (12)	−0.0024 (12)	0.0047 (10)	0.0001 (12)
C5	0.0369 (14)	0.0390 (17)	0.0428 (15)	0.0014 (13)	−0.0008 (12)	−0.0081 (13)
C6	0.0404 (16)	0.0428 (19)	0.0500 (17)	−0.0026 (14)	−0.0078 (13)	−0.0094 (14)
C7	0.0303 (13)	0.0460 (18)	0.0491 (15)	−0.0025 (13)	0.0011 (11)	−0.0001 (14)
C8	0.0324 (13)	0.0398 (18)	0.0432 (14)	0.0010 (13)	0.0022 (11)	−0.0012 (13)
C8A	0.0324 (13)	0.0338 (16)	0.0336 (13)	0.0001 (12)	0.0049 (10)	−0.0004 (12)
C9	0.0420 (15)	0.0328 (17)	0.0509 (16)	0.0027 (13)	−0.0023 (12)	−0.0047 (13)
C10	0.0468 (16)	0.0366 (18)	0.0512 (16)	0.0047 (14)	−0.0100 (13)	−0.0096 (14)
C10A	0.0345 (13)	0.0347 (16)	0.0429 (15)	−0.0011 (12)	0.0018 (11)	−0.0053 (12)
C11	0.069 (2)	0.053 (2)	0.061 (2)	0.0181 (18)	−0.0263 (16)	−0.0213 (18)
C12	0.122 (4)	0.091 (3)	0.057 (2)	−0.063 (3)	−0.021 (2)	0.000 (2)
C13	0.056 (2)	0.129 (4)	0.060 (2)	−0.009 (2)	0.0117 (16)	−0.046 (3)
C14	0.0359 (14)	0.070 (2)	0.0441 (15)	0.0045 (16)	0.0121 (12)	0.0086 (16)
C15	0.0504 (17)	0.046 (2)	0.0610 (19)	0.0002 (15)	−0.0043 (14)	0.0134 (16)
C16	0.0399 (16)	0.076 (3)	0.071 (2)	0.0171 (18)	0.0046 (15)	−0.018 (2)
C17	0.0321 (13)	0.0477 (19)	0.0441 (16)	0.0019 (13)	0.0061 (11)	0.0081 (14)
C18	0.0345 (13)	0.0351 (16)	0.0391 (14)	−0.0002 (12)	0.0033 (11)	−0.0020 (12)
C19	0.0370 (14)	0.0466 (18)	0.0426 (15)	0.0003 (14)	0.0040 (12)	−0.0001 (14)
C20	0.0525 (18)	0.065 (2)	0.0442 (15)	0.0038 (17)	0.0011 (14)	0.0082 (16)
C21	0.0441 (16)	0.052 (2)	0.0562 (18)	0.0095 (16)	−0.0109 (14)	−0.0003 (16)
C22	0.0340 (15)	0.058 (2)	0.0602 (19)	0.0077 (15)	0.0039 (13)	−0.0046 (17)
C23	0.0344 (14)	0.055 (2)	0.0471 (15)	0.0033 (14)	0.0057 (12)	0.0011 (15)

### Geometric parameters (Å, º)

**Table:**

O1—C17	1.374 (3)	C10—C10A	1.520 (4)
O1—C2	1.422 (3)	C10—H10A	0.9900
O2—C17	1.192 (3)	C10—H10B	0.9900
C1—C2	1.381 (4)	C11—C12	1.524 (6)
C1—C10A	1.417 (4)	C11—C13	1.524 (6)
C1—C11	1.528 (4)	C11—H11	1.0000
C2—C3	1.377 (4)	C12—H12A	0.9800
C3—C4	1.378 (4)	C12—H12B	0.9800
C3—H3	0.9500	C12—H12C	0.9800
C4—C4A	1.390 (4)	C13—H13A	0.9800
C4—H4	0.9500	C13—H13B	0.9800
C4A—C10A	1.402 (4)	C13—H13C	0.9800
C4A—C4B	1.542 (3)	C14—H14A	0.9800
C4B—C5	1.540 (4)	C14—H14B	0.9800
C4B—C14	1.547 (4)	C14—H14C	0.9800
C4B—C8A	1.550 (4)	C15—H15A	0.9800
C5—C6	1.523 (4)	C15—H15B	0.9800
C5—H5A	0.9900	C15—H15C	0.9800
C5—H5B	0.9900	C16—H16A	0.9800
C6—C7	1.518 (4)	C16—H16B	0.9800
C6—H6A	0.9900	C16—H16C	0.9800
C6—H6B	0.9900	C17—C18	1.474 (4)
C7—C8	1.528 (4)	C18—C19	1.387 (4)
C7—H7A	0.9900	C18—C23	1.392 (4)
C7—H7B	0.9900	C19—C20	1.380 (4)
C8—C15	1.528 (4)	C19—H19	0.9500
C8—C16	1.531 (4)	C20—C21	1.390 (5)
C8—C8A	1.557 (4)	C20—H20	0.9500
C8A—C9	1.518 (4)	C21—C22	1.369 (5)
C8A—H8A	1.0000	C21—H21	0.9500
C9—C10	1.518 (4)	C22—C23	1.378 (4)
C9—H9A	0.9900	C22—H22	0.9500
C9—H9B	0.9900	C23—H23	0.9500
			
C17—O1—C2	116.01 (19)	C10A—C10—H10B	108.5
C2—C1—C10A	117.7 (2)	H10A—C10—H10B	107.5
C2—C1—C11	121.2 (3)	C4A—C10A—C1	120.6 (3)
C10A—C1—C11	121.2 (3)	C4A—C10A—C10	120.4 (2)
C3—C2—C1	122.8 (2)	C1—C10A—C10	119.0 (2)
C3—C2—O1	117.6 (2)	C12—C11—C13	110.5 (3)
C1—C2—O1	119.6 (2)	C12—C11—C1	114.6 (3)
C2—C3—C4	118.7 (3)	C13—C11—C1	112.1 (3)
C2—C3—H3	120.7	C12—C11—H11	106.3
C4—C3—H3	120.7	C13—C11—H11	106.3
C3—C4—C4A	121.8 (2)	C1—C11—H11	106.3
C3—C4—H4	119.1	C11—C12—H12A	109.5
C4A—C4—H4	119.1	C11—C12—H12B	109.5
C4—C4A—C10A	118.5 (2)	H12A—C12—H12B	109.5
C4—C4A—C4B	118.9 (2)	C11—C12—H12C	109.5
C10A—C4A—C4B	122.6 (2)	H12A—C12—H12C	109.5
C5—C4B—C4A	110.8 (2)	H12B—C12—H12C	109.5
C5—C4B—C14	108.4 (2)	C11—C13—H13A	109.5
C4A—C4B—C14	105.85 (19)	C11—C13—H13B	109.5
C5—C4B—C8A	108.6 (2)	H13A—C13—H13B	109.5
C4A—C4B—C8A	108.4 (2)	C11—C13—H13C	109.5
C14—C4B—C8A	114.9 (2)	H13A—C13—H13C	109.5
C6—C5—C4B	113.1 (2)	H13B—C13—H13C	109.5
C6—C5—H5A	109.0	C4B—C14—H14A	109.5
C4B—C5—H5A	109.0	C4B—C14—H14B	109.5
C6—C5—H5B	109.0	H14A—C14—H14B	109.5
C4B—C5—H5B	109.0	C4B—C14—H14C	109.5
H5A—C5—H5B	107.8	H14A—C14—H14C	109.5
C7—C6—C5	111.0 (2)	H14B—C14—H14C	109.5
C7—C6—H6A	109.4	C8—C15—H15A	109.5
C5—C6—H6A	109.4	C8—C15—H15B	109.5
C7—C6—H6B	109.4	H15A—C15—H15B	109.5
C5—C6—H6B	109.4	C8—C15—H15C	109.5
H6A—C6—H6B	108.0	H15A—C15—H15C	109.5
C6—C7—C8	113.5 (2)	H15B—C15—H15C	109.5
C6—C7—H7A	108.9	C8—C16—H16A	109.5
C8—C7—H7A	108.9	C8—C16—H16B	109.5
C6—C7—H7B	108.9	H16A—C16—H16B	109.5
C8—C7—H7B	108.9	C8—C16—H16C	109.5
H7A—C7—H7B	107.7	H16A—C16—H16C	109.5
C15—C8—C7	110.4 (2)	H16B—C16—H16C	109.5
C15—C8—C16	107.4 (3)	O2—C17—O1	122.5 (2)
C7—C8—C16	107.5 (2)	O2—C17—C18	125.0 (2)
C15—C8—C8A	114.1 (2)	O1—C17—C18	112.4 (2)
C7—C8—C8A	108.4 (2)	C19—C18—C23	119.5 (3)
C16—C8—C8A	108.7 (2)	C19—C18—C17	123.3 (2)
C9—C8A—C4B	109.0 (2)	C23—C18—C17	117.2 (2)
C9—C8A—C8	114.9 (2)	C20—C19—C18	120.3 (3)
C4B—C8A—C8	116.9 (2)	C20—C19—H19	119.9
C9—C8A—H8A	104.9	C18—C19—H19	119.9
C4B—C8A—H8A	104.9	C19—C20—C21	119.5 (3)
C8—C8A—H8A	104.9	C19—C20—H20	120.2
C10—C9—C8A	111.3 (3)	C21—C20—H20	120.2
C10—C9—H9A	109.4	C22—C21—C20	120.4 (3)
C8A—C9—H9A	109.4	C22—C21—H21	119.8
C10—C9—H9B	109.4	C20—C21—H21	119.8
C8A—C9—H9B	109.4	C21—C22—C23	120.3 (3)
H9A—C9—H9B	108.0	C21—C22—H22	119.8
C9—C10—C10A	115.0 (2)	C23—C22—H22	119.8
C9—C10—H10A	108.5	C22—C23—C18	120.0 (3)
C10A—C10—H10A	108.5	C22—C23—H23	120.0
C9—C10—H10B	108.5	C18—C23—H23	120.0
